# The State Lunatic Asylum, New York. (With an Engraving.)

**Published:** 1850-10-01

**Authors:** 


### THE STATE LUNATIC ASYLUM, NEW YORK.

With this number we give an engraving of this national institution. The building was commenced in 1839, and finished in 1842. It is situated about a mile from the city of Utica, and 240 miles from New York. It is built upon an elevated plane, commanding a fine view of Utica, and the hill in the direction of the Trenton Falls. The Erie Canal runs a short distance in front of the Asylum. It is built of hewn limestone, in the Doric style of architecture. In the centre of the building is the superintendent's office, and a room for the reception of visitors. The asylum is divided into two wings for male and female patients. The wings are 215 feet in length, having a spacious hall of 13 feet in width the entire length, with rooms on each side for the use of patients. Each hall is occupied by 35 or 40 patients, composing one family, under the care of attendants, and having no necessary connexion with any other part of the house. There are also wings in the rear of the asylum. The building is so constructed as to admit of the patients being divided into 12 different classes, each class occupying separate apartments. There are 380 single rooms for patients; 24 for attendants; 20 associated dormitories, that will accommodate from 5 to 12 persons; 16 parlour or day rooms; 12 dining rooms; 24 bathing rooms, and the same number of water-closets. The apartments are warmed by hot air. There is associated with the Asylum 133 acres of fertile and productive farm-land. We have no report of this Asylum since 1847; in that year there were in the institution 802 patients, 406 men, and 396 women. In the preceding year there was discharged 330 patients, the recoveries amounting to 187. Since the opening of the asylum, there have been admitted 1609 patients; discharges, 1,137; recovered, 640; improved, 269; unimproved, 114; died, 114. There are 46 persons exclusively employed as attendants in the Asylum. The whole institution consists of 570 persons. In *one week* the amount of provisions consumed was as follows:—wheat flower, 16 barrels; 1800lb. of fresh and corned beef and mutton; 280lb. of salt fish; butter, 650lb.; potatoes, 30 bushels. In addition to this, there are consumed a considerable quantity of Indian meal, buckwheat, poultry, tea, coffee, cheese, milk, and sugar. The number of visitors in one year amounted to 4229! We propose giving in a future number a more full description of the management of this Asylum, over which the late Dr. Brigham for so many years presided. We subjoin one of the theatrical bills of the Asylum:—

### ASYLUM THEATRE.

*Great Bill for Tuesday Evening, Nov. 30.—4th performance of this season.*

---

The entertainment will commence with an original play in three acts, entitled

MORE WEIGHT THAN BUTTER.

Mr. Davis, (Plaintiff) .............................. Mr. J. M. B.
" Andrews,.............................................. " W. K.
Judges,...................... Messrs. H. G. R., A. G. S. M., and J. C. F.
Scriptus Ekreton, (Clerk of Court,) ..................... Mr. W. W. W.

Mr. Frost, (Constable,) .................................. Mr. E. P., Jr.
Grand Jury, ....................... Messrs. J. C. M., J. C. G., & R. M.
Jury, ........Messrs. J. C. G., D. L., G. W. B., C. W., R. W. P., & R. M.
Spectators,........... Messrs. D. S., J. R., R. W. P., D. L., C. W., & G. B.
Mrs. Andrews, (Defendant,)...................... Mr. D. A. S.
Mrs. Davis,.................................. " J. R.

SONGS AND RECITATIONS.

Go Ahead, ........................................... Mr. A. J. D.
Virtues of Tea, ........................................ " P. M.
Song—The Raging Ca-nawl, ........................... " J. M. B.
The Sailor Boy, ........................................ " G. W. W.
Young Farmer "Abroad," and Canal Boy at "Home," Messrs. J. R. & D. A. S.

After which, will be performed a Comic Impromptu, entitled

PAYING FOR ADVICE.

Timothy Tarbox, .................................... Mr. J. M. B.
Dr. Wiseacre,........................................ " D. L.
Lawyer Doolittle, ....................................... " D. A. S.
Judge Hawthorn, ........................................ " J. C. M.

SONGS AND RECITATIONS.

Lodgings to Let, .................................... Mr. H. De M.
Comic Duet, ............................ Messrs. J. M. B. & M. G. P.
The Fallen Brave, .................................. Mr. A. G. S. M.
Grave of Bonaparte, ............. Messrs. C. W., D. S., J. R., & M. G. P.

Following which, will be presented

GENIUS *versus* VULGARITY.

Mr. Leonardo, (Artist,) .................................. Mr. G. W. B.
Capt. Jones, (Innkeeper,) ................................ " J. C. M.
Jonathan, (a Yankee,)....................................... " J. R.
Mr. Alphonso Montague, (an Exquisite,) ................. " G. W. P.

The Beggar's Petition,................................... Mr. P. M.
Music—Hunting Piece, ..................... C. W., J. R., & E. P., Jr.
Genevera, ........................................... Mr. M. G. P.

The whole to conclude with

ETHIOPIAN SAYINGS AND DOINGS.

*The Curtain will rise at half-past 7 o'clock, precisely.*

[Printed at the Asylum.]

